# Efficient Non-Doped White Organic Light-Emitting Diodes Based on an Interface Exciplex Structure

**DOI:** 10.3390/mi17080940

**Published:** 2026-08-07

**Authors:** Qing Zhao, Ping Chen, Tianyu Zhang

**Affiliations:** 1 Key Laboratory of Geophysical Exploration Equipment, Ministry of Education, College of Instrumentation and Electrical Engineering, Jilin University, Changchun 130012, China; zhaoqing24@mails.jlu.edu.cn; 2 Institute of Physics and Electronic Information, Yantai University, Yantai 264005, China

**Keywords:** high-efficiency, non-doped, OLEDs, interface exciplex structure

## Abstract

Non-doped organic light-emitting diodes (OLEDs) feature simple fabrication processes and favorable repeatability, yet their overall device performance remains unsatisfactory. Here we propose a novel white OLED based on ultrathin emissive layers (EMLs). The novel efficient device structure is constructed based on an interface exciplex structure. Color-stable or color-tunable white OLEDs can be realized by tuning the interlayer thickness. The resulting color-tunable white device obtains a maximum power efficiency of 32.03 lm/W with a correlated color temperature span from 4091 K to 5376 K. Furthermore, the luminescence mechanism is thoroughly investigated. The shift of the spectra in the color-tunable device is mainly attributed to the movement of the main exciton recombination zone.

## 1. Introduction

White organic light-emitting diodes (OLEDs) have attracted much attention in the display and lighting fields because of their high efficiency, flexibility and lightweight nature [1,2,3,4,5]. Various strategies have been proposed to develop high-performance white OLEDs such as adopting multiple emissive layers (EMLs), a single EML doped with different dyes, or tandem structure [6,7,8]. Although white OLEDs fabricated using a doping method have demonstrated high device performance, they still face limitations in device design and fabrication.

Exciplex structures are capable of efficiently harvesting triplet excitons via the reverse intersystem crossing (RISC) process, thereby enabling a theoretical internal quantum efficiency approaching 100% [9,10,11,12]. The interface exciplex structure eliminates the requirement for co-doping of donor and acceptor materials during fabrication, which simplifies the manufacturing process [13,14]. Moreover, such structures have been verified to deliver performance comparable to that of conventional exciplex host systems. In 2017, Xu et al. fabricated a green phosphorescent device based on an interface exciplex formed by TAPC and 1,3,5-tri(p-pyrid-3-ylphenyl)benzene [15]. The maximum external quantum efficiency (EQE) of 17.97% and current efficiency of 66.2 cd/A of the green device were obtained. Dong et al. reported a high-efficiency and color-stable hybrid white device based on an interface exciplex [16]. The resulting device with a doped blue EML where a blue TADF dye was doped into the donor materials emitted warm white light with an EQE of 22.3%. Non-doped white OLEDs have attracted increasing research interest in recent years owing to their simplified device architectures and low-cost manufacturing potential, rendering them promising candidates for high-efficiency white devices [17,18,19]. Nevertheless, few studies to date have reported non-doped white OLEDs constructed by integrating interfacial exciplex structures with ultrathin EMLs [20].

In this work, we propose an innovative design strategy for non-doped white OLEDs relying on interfacial exciplex structures. Unlike previously reported studies, the device architecture developed herein eliminates the need for an extra interlayer. Either the donor or acceptor material serves simultaneously as an interlayer to tune the emission color, thereby simplifying the device configuration. By inserting blue and orange phosphorescent ultrathin EMLs at the 1,3-Bis(carbazol-9-yl)benzene (MCP)/4,6-Bis(3,5-di-4-pyridinylphenyl)-2-phenylpyrimidine (B4PyPPM) interfaces, a series of non-doped two-color white OLEDs were fabricated. Color-stable or color-tunable white OLEDs can be realized by tuning the interlayer thickness. The fabricated color-tunable white device achieves a peak power efficiency of 32.03 lm/W, with its correlated color temperature varying from 4091 K to 5376 K. Furthermore, the underlying luminescence mechanism is systematically analyzed. This work demonstrates an effective strategy for simplifying the structure of non-doped white OLEDs, providing useful guidance for the development of high-efficiency, low-cost non-doped white OLEDs.

## 2. Materials and Methods

**Figure 1:**
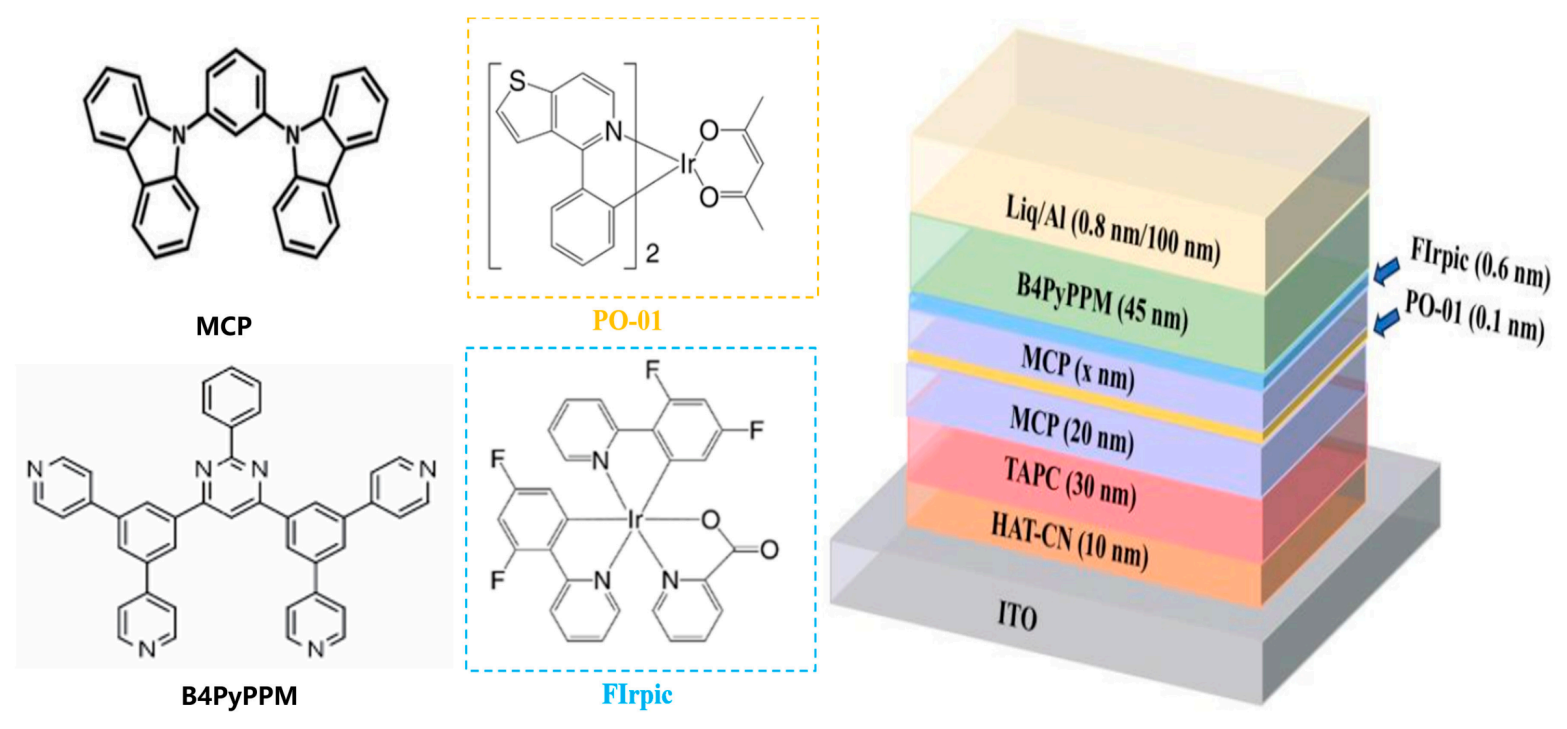
Molecular structures of the organic materials used and a schematic diagram of the proposed devices.

The devices were fabricated by thermal evaporation under high vacuum of 3.0 × 10^−4^ Pa on ITO-coated glass substrates with a sheet resistance of 10 Ω/sq. HAT-CN and TAPC served as the hole injection layer and hole transport layer (HTL), respectively. B4PyPPM was used as electron transport layer (ETL). MCP and B4PyPPM were selected as the donor and the acceptor to form interface exciplex. We used PO-01 and FIrpic as orange and blue phosphorescent dyes. A 0.8 nm Liq layer covered by 100 nm Al was used as the cathode. The deposition rates of organic and metal materials were kept at 0.05–0.3 Å/s and 0.1–0.5 nm/s, respectively. The Commission Internationale de l’Eclairage (CIE) chromaticity coordinates, electroluminescent (EL) spectra, and luminance data were recorded simultaneously via a SpectraScan photometer (SpectraScan PR-655, Photo Research, USA). Current density, current efficiency, power efficiency, and EQE curves were measured using a source-measure unit (Model 2400, Keithley, a Tektronix company, Solon, OH, USA) combined with an absolute test system. All device characterizations were performed at room temperature under ambient atmosphere without encapsulation. All organic materials were commercially obtained from Shenzhen PURI Materials Technologies Co., Ltd. (Shenzhen, China). The molecular structures of the used phosphorescent dyes and the schematic diagram of the proposed devices are depicted in Figure 1.

## 3. Results

To fabricate high-efficiency non-doped white OLEDs, we constructed a set of devices utilizing an MCP/B4PyPPM interfacial exciplex with the following architecture: ITO/HAT-CN (10 nm)/TAPC (30 nm)/MCP (20 nm)/PO-01 (0.1 nm)/MCP (x nm)/FIrpic (0.6 nm)/B4PyPPM (45 nm)/Liq (0.8 nm)/Al (100 nm). An MCP interlayer of variable thickness x (0, 3, 5, 7 nm for devices W1–W4) is inserted between the blue and orange ultrathin EMLs to modulate the dominant exciton recombination zone and energy transfer.

**Figure 2:**
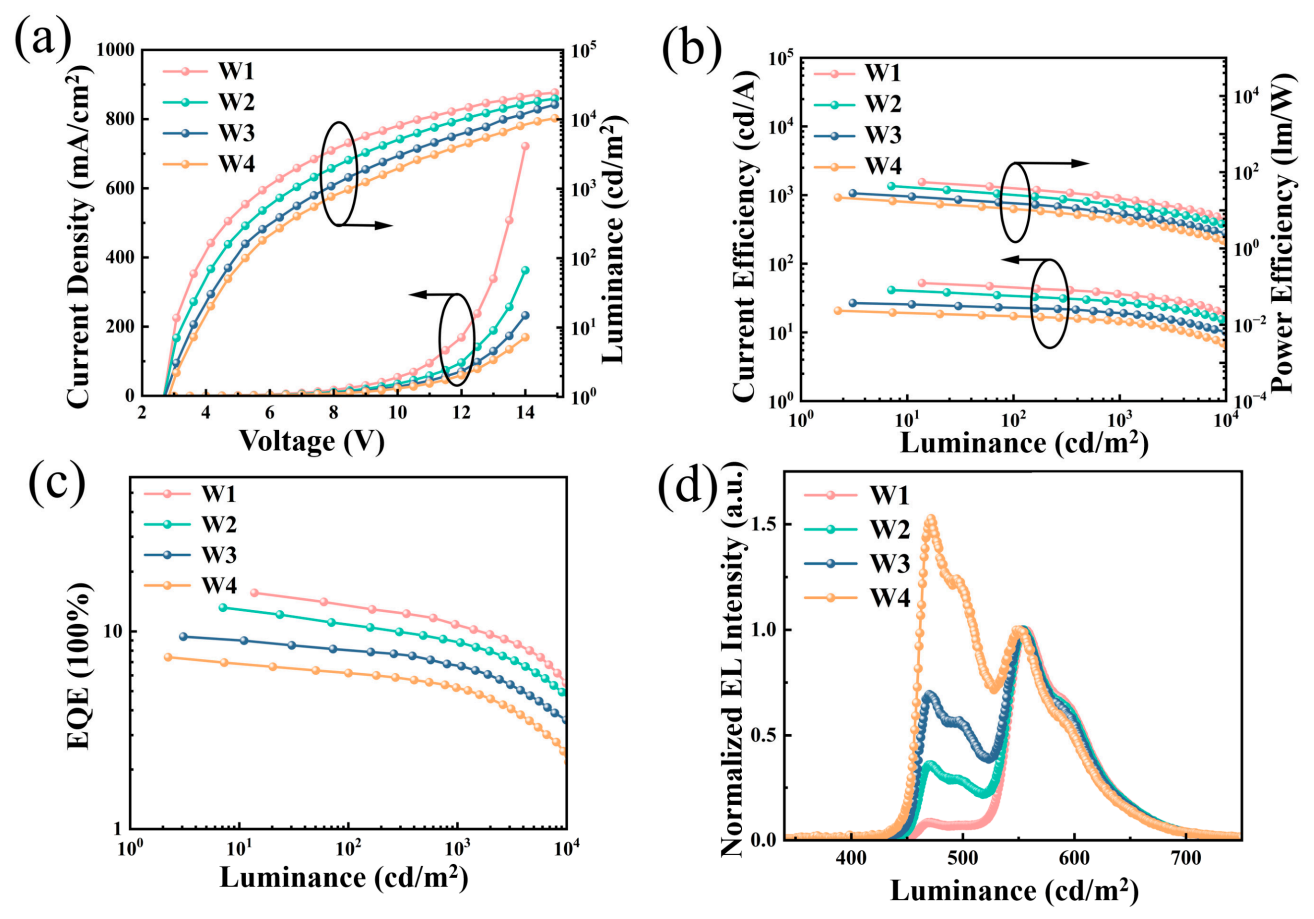
Current density–voltage–luminance characteristics (**a**), current efficiency–luminance–power efficiency characteristics (**b**), EQE–luminance characteristics (**c**), and normalized EL spectra at 8 V (**d**) of devices W1–W4.

Figure 2a shows the current density–voltage–luminance characteristics of devices W1–W4. As the MCP interlayer thickness rises, the current density of the devices gradually drops under identical applied voltages. Notably, all devices exhibit a low turn-on voltage of merely 2.6 V. As the MCP interlayer thickness increases, the maximum luminance gradually declines from 26,879 cd/m^2^ to 13,254 cd/m^2^. The current efficiency–luminance–power efficiency and EQE–luminance characteristics are shown in Figure 2b and Figure 2c, respectively. Device W1 delivers the optimal performance, with peak efficiencies reaching 52.38 cd/A, 54.85 lm/W, and 15.64%. In contrast, the corresponding values of device W4 drop to 20.71 cd/A, 21.68 lm/W, and 7.41%. As the thickness of MCP increases, energy transfer from FIrpic to PO-01 becomes less efficient. The suppression of high-efficiency orange emission from PO-01 leads to deteriorated EL performance of the device. Furthermore, severe exciton annihilation in the blue emitter FIrpic also impairs the luminescence performance [21].

The normalized EL spectra at 8 V are shown in Figure 2d. The spectra of the four devices show two distinct emission peaks at 471 nm and 553 nm, which correspond to the emission of FIrpic and PO-01, respectively. As the MCP interlayer thickness increases, the intensity of the blue emission rises steadily, while the orange emission intensity declines gradually. Correspondingly, the CIE chromaticity coordinates shift from (0.45, 0.51) for device W1 to (0.27, 0.40) for device W4. Despite delivering the maximum efficiency, device W1 shows overly orange-shifted emission, which is unfavorable for balanced white light output. In comparison, device W3 maintains relatively high efficiency and possesses CIE coordinates of (0.35, 0.45), rendering it a promising candidate for further optimization toward high-quality white emission.

For devices W1–W4, exciton recombination takes place at the MCP/B4PyPPM interface. High exciton density in this narrow interface greatly degrades luminescence performance. Inserting a B4PyPPM interlayer next to the PO-01 ultrathin EML generates two exciton recombination zones and improves exciton utilization efficiency. To achieve non-doped white OLEDs with higher EL efficiency, another series of non-doped white devices is further constructed with the structure ITO/HAT-CN (10 nm)/TAPC (30 nm)/MCP (20 nm)/PO-01 (0.15 nm)/B4PyPPM (y nm)/MCP (5 nm)/FIrpic (0.6 nm)/B4PyPPM (45 nm)/Liq (0.8 nm)/Al (100 nm), where y = 1, 3, and 4, corresponding to devices W5–W7, respectively.

**Figure 3:**
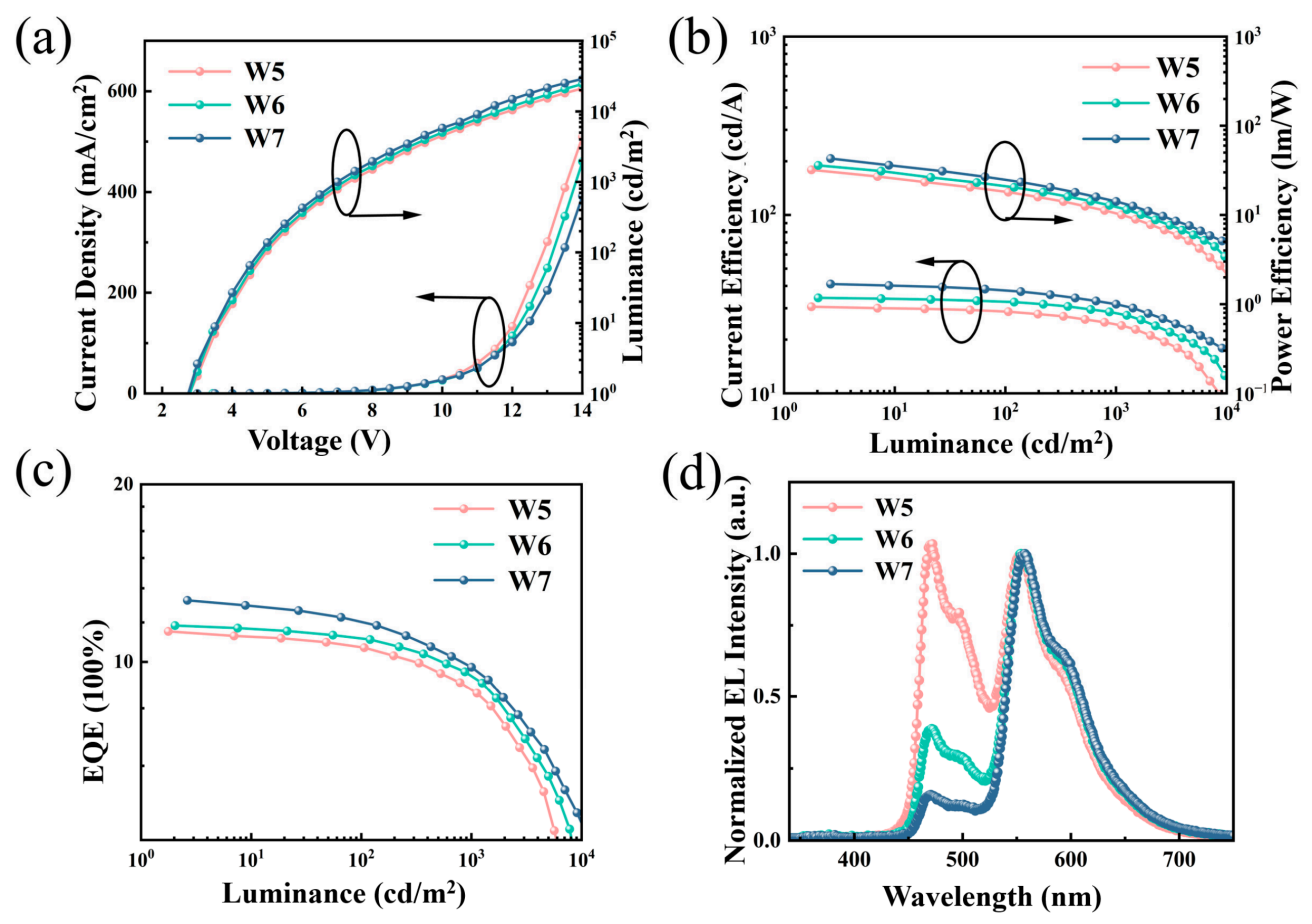
Current density–voltage–luminance characteristics (**a**), current efficiency–luminance–power efficiency characteristics (**b**), EQE–luminance characteristics (**c**), and normalized EL spectra of devices W5–W7 at 8 V (**d**).

**Figure 4:**
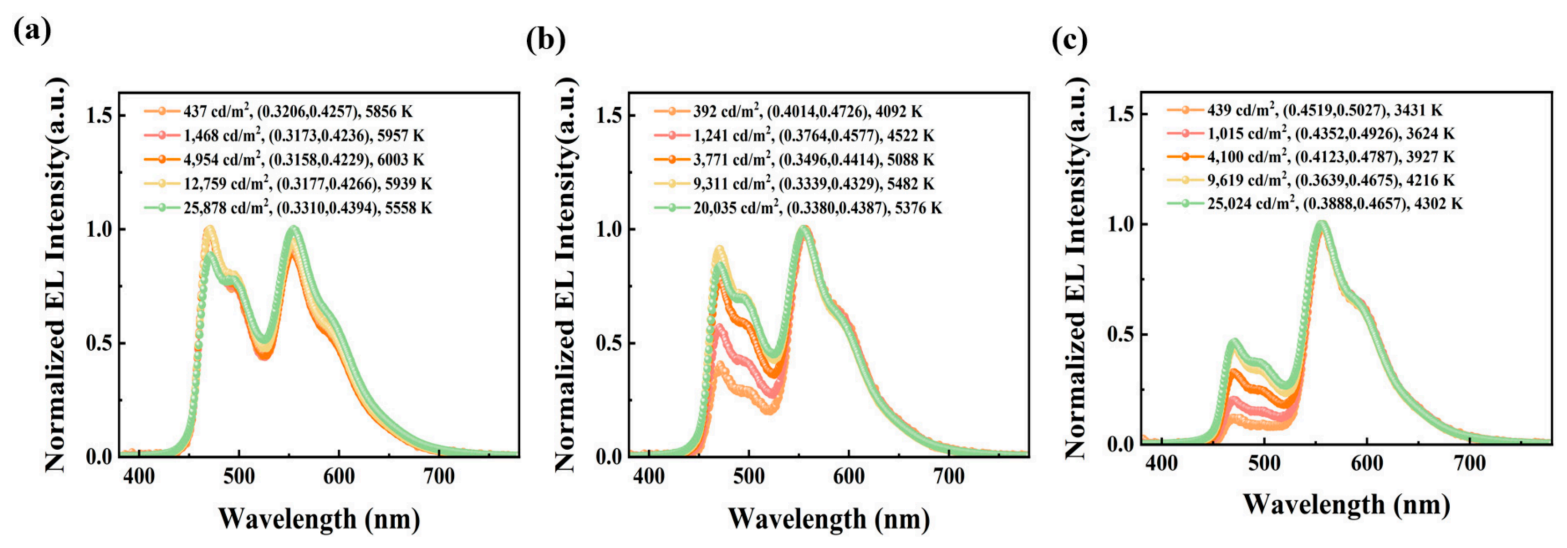
Normalized EL spectra of devices (**a**) W5, (**b**) W6 and (**c**) W7 at different luminance.

The current density–voltage–luminance characteristic of devices W5–W7 are shown in Figure 3a. All devices feature a low turn-on voltage of approximately 2.7 V, demonstrating that the MCP/B4PyPPM interfacial exciplex facilitates charge injection, charge transport, and exciton generation. Table 1 summarizes the detailed performance of devices W1–W7. As the thickness of the B4PyPPM interlayer increases, the peak luminance increases from 24,774 cd/m^2^ (device W5) to 32,636 cd/m^2^ (device W7), alongside a slight reduction in current density. Figure 4b,c reveal that device efficiencies gradually improve with thicker B4PyPPM layers. When the B4PyPPM thickness is tuned from 1 nm to 4 nm, the maximum current efficiency, power efficiency, and EQE increase correspondingly from 30.59 cd/A, 32.03 lm/W, and 11.27% for device W5 to 41.03 cd/A, 42.96 lm/W, and 12.72% for device W7, respectively. These observations confirm that adjusting the B4PyPPM thickness within a proper range optimizes interfacial exciton dynamics and elevates overall device performance. The efficiencies of device W5 are even comparable to most reported hybrid WOLEDs employing doped EMLs, as summarized in Table S1 in in the Supplementary Materials. Although device W7 achieves the highest efficiency, device W5 displays far more stable EL spectra and delivers better color balance compared with device W7, rendering it more promising for lighting applications. It should be noted that all devices display obvious efficiency roll-off. Taking device W5 as an example, an efficiency roll-off of 20.6% is observed at a luminance of 1000 cd/m^2^. Such efficiency roll-off mainly originates from exciton annihilation within the ultrathin blue EML.

The normalized EL spectra shown in Figure 3d reveal that the blue emission intensity gradually decreases, whereas the orange emission gradually increases, as the B4PyPPM interface layer becomes thicker. The CIE coordinates of devices W5–W7 at 8 V are (0.32, 0.42), (0.40, 0.47), and (0.44, 0.49), respectively, all of which fall within the white emission region. However, the emission color progressively shifts toward the warm white region as the B4PyPPM thickness increases.

The normalized EL spectra of devices W5–W7 at different luminance levels are shown in Figure 4. It is noteworthy that device W5 exhibits excellent spectral stability. As the luminance varies from 437.9 cd/m^2^ to 25,878.5 cd/m^2^, the CIE coordinates shift only slightly from (0.32, 0.43) to (0.33, 0.44). Note that the intensity of the blue emission gradually increases relative to that of the orange emission in device W6 and device W7. The shift in spectra is mainly attributed to the change in the dominant emission route with the increasing luminance. For device W6, the CIE coordinates shift from (0.40, 0.47) to (0.34, 0.44) as the luminance increases from 392.0 cd/m^2^ to 20,035.9 cd/m^2^, and the correlated color temperature (CCT) rises from 4091 K to 5376 K, corresponding to a CCT span of 1285 K.

**Figure 5:**
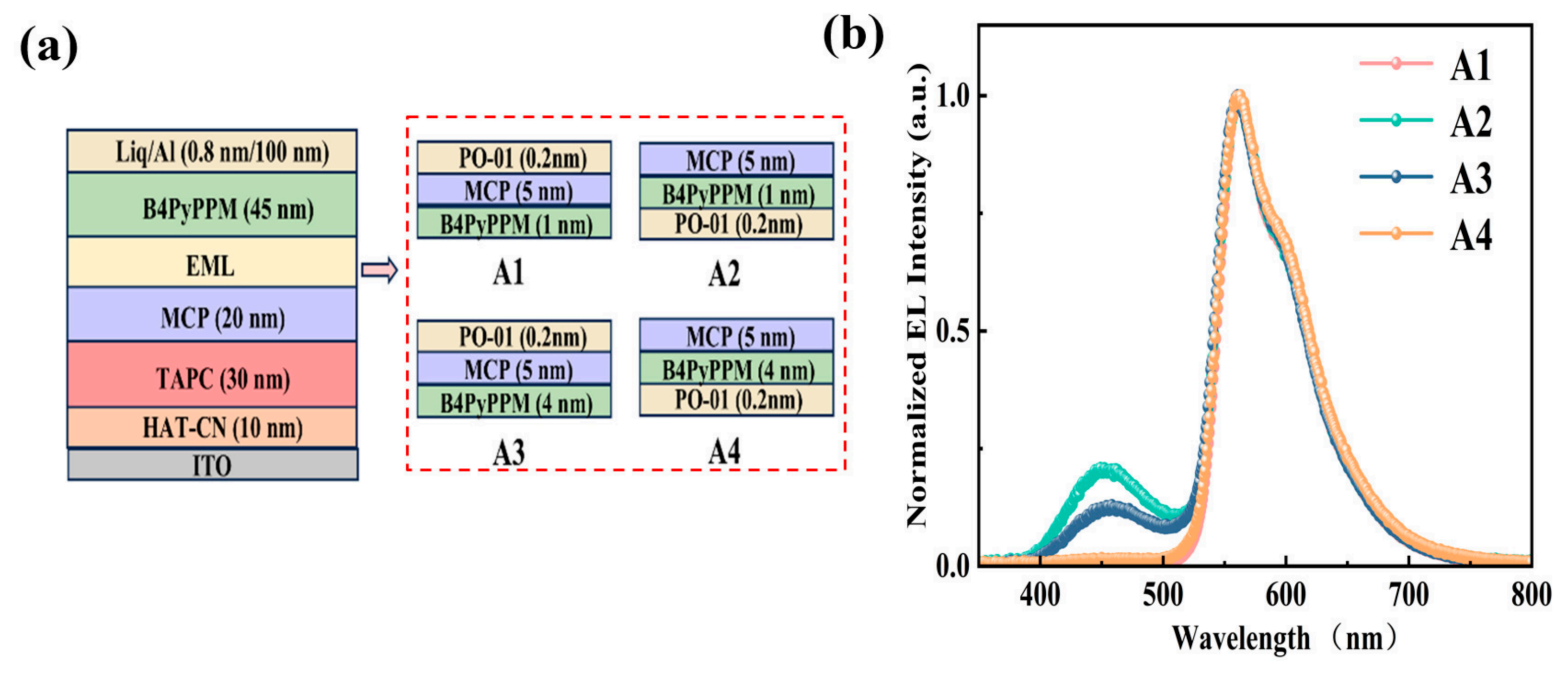
Device structures of A1–A4 (**a**) and normalized EL spectra at 8 V (**b**).

We have also investigated the main recombination zone in devices W5–W7. To identify the main recombination zone, PO-01 is used as a phosphorescent sensing dopant and inserted at different positions near the interface. The structures of the probe devices are shown in Figure 5a. Figure 5b shows the EL spectra of devices A1–A4. When the B4PyPPM thickness is 1 nm, device A1 exhibits the characteristic emission peak of PO-01 at 563 nm, indicating complete energy transfer from the interface exciplex to PO-01. In contrast, device A2 exhibits two emission peaks at 452 nm and 563 nm, indicating incomplete energy transfer and suggesting that the main recombination zone is located near the interface between the 5 nm MCP layer and ETL. When the B4PyPPM thickness increases to 4 nm, device A3 shows two emission peaks at 452 and 563 nm, whereas device A4 retains only the 563 nm peak. The observation indicates that the main recombination zone shifts toward the HTL side. Therefore, increasing the thickness of the interlayer B4PyPPM causes the main recombination zone to gradually move from the ETL side to the hole-transport-layer side. For device W5, the 1 nm thick B4PyPPM interlayer barely affects carrier transport and exciton formation. Combined with probing experiments, the main exciton recombination zone of device W5 is located at the interface between 5 nm MCP and the ETL. Interface exciplex transfers energy to the ultrathin EML via stable energy transfer, so device W5 behaves similarly to conventional single-EML devices in which the exciton recombination zone remains stationary and thus yields highly stable emission spectra [18]. In contrast, increasing the thickness of the B4PyPPM interlayer introduces an additional interface, where carrier accumulation generates another exciton recombination zone. Accordingly, devices W6 and W7 resemble conventional white OLEDs with two separate EMLs. As the applied voltage increases, more holes overcome the injection barrier of the B4PyPPM interlayer, and the exciton recombination zone gradually shifts toward the blue-emitting region, facilitating enhanced blue emission [22].

**Figure 6:**
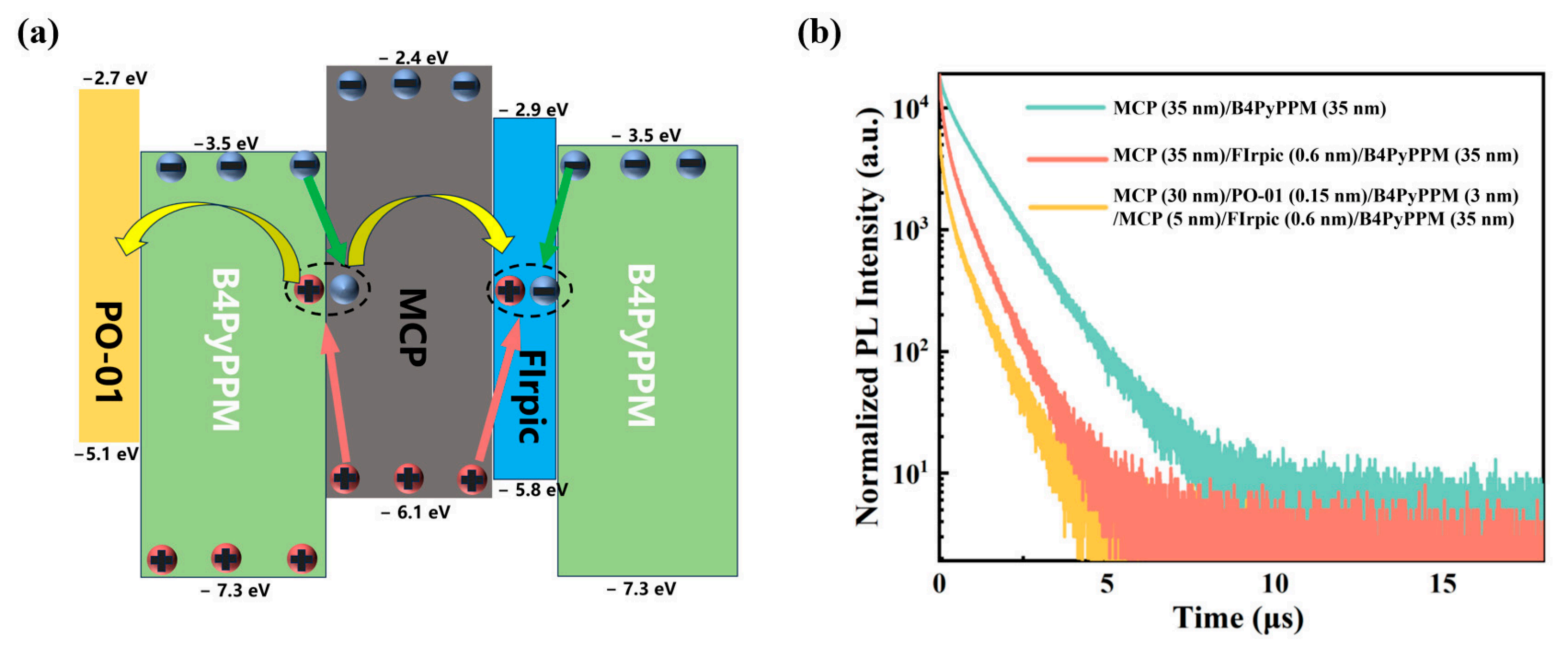
Emission mechanism diagram (**a**) for the proposed devices, and the transient PL decay of mixed films measured at 448 nm (**b**).

Figure 6a illustrates the emission mechanism of device W5, with energy levels of constituent materials adopted from the previously reported literature [23]. As illustrated, excitons formed at the MCP/B4PyPPM interface transfer energy to the blue- and orange-emitting layers through Förster energy transfer. As the driving voltage increases, an increasing number of holes accumulate at the interface adjacent to ETL, which shifts the exciton recombination zone toward the ETL and consequently enables tunable emission color. The better performance of device W5 should be due to the efficient RISC property of the exciplex, which promotes Förster energy transfer mechanism and maximizes exciton utilization, helping to improve the device efficiency [24]. As shown in Figure 6b, the pure interface exciplex film displays the longest lifetime among the three transient PL decay profiles. The lifetimes of the films incorporating blue and orange ultrathin EMLs gradually decrease, which indicates that Förster energy transfer between the interface exciplex and dyes is progressively enhanced. Furthermore, we calculate the energy transfer rate of the Förster energy transfer. The calculated rate is 5.3 × 10^6^ s^−1^ after the introduction of both blue and orange EMLs, indicating efficient Förster energy transfer from the interface exciplex to blue and orange ultrathin EMLs indeed occurs.

## 4. Discussion

By adopting the interfacial exciplex architecture, we designed two types of white OLEDs with stable emission spectra and tunable CCT. Owing to the limitation imposed by the single interface exciplex configuration adopted in this work, the resulting devices exhibit a slightly increased turn-on voltage. As illustrated in Figure 6a, hole transport from MCP to B4PyPPM requires overcoming an interfacial energy barrier of up to 1.2 eV, while electrons moving from B4PyPPM to MCP face a barrier of 1.1 eV. Adopting bulk exciplex architectures or choosing material systems with smaller interfacial energy barriers represents an effective approach to reduce the turn-on voltage. Moreover, white OLEDs with prolonged operational lifetime are expected to be realized when more stable TADF or fluorescent emitters are utilized as blue materials. We believe this research offers a valuable guideline for the rational design of non-doped white OLEDs.

## 5. Conclusions

Color-stable or color-tunable white OLEDs were realized via tuning the interlayer thickness. The fabricated color-tunable white device reaches a peak power efficiency of 32.03 lm/W, with its correlated color temperature (CCT) ranging from 4091 K to 5376 K. The high performance of these devices can be attributed to the RISC process originating from interface exciplexes. The variation in CCT of the color-tunable OLEDs mainly arises from the shift in the dominant exciton recombination zone. Furthermore, the luminescence mechanism of the devices was discussed in detail. This work provides a feasible reference strategy for the design of high-performance non-doped OLEDs.

## Figures and Tables

**Table 1:** Detailed performance data of devices W1–W7.

Device	V_on_ ^a^ (V)	L_max_ ^b^ (cd/m^2^)	CE_max/1000_ ^b/c^ (cd/A)	PE_max_ ^b^ (lm/W)	EQE_max_ ^b^ (%)	CIE ^d^
W1	2.6	26,879	52.38/36.30	54.85	15.64	(0.45, 0.51)
W2	2.6	21,578	41.38/27.85	43.33	13.18	(0.40, 0.47)
W3	2.6	19,913	26.79/19.01	28.05	9.41	(0.35, 0.45)
W4	2.6	13,254	20.71/14.55	21.68	7.41	(0.27, 0.40)
W5	2.7	24,774	30.59/24.30	32.03	11.27	(0.32, 0.42)
W6	2.7	28,647	34.30/27.73	35.92	11.52	(0.40, 0.47)
W7	2.7	32,636	41.03/32.09	42.96	12.72	(0.44, 0.49)

^a^ At the luminance of 1 cd/m^2^; ^b^ maximum luminance; ^c^ measured at 1000 cd/m^2^; ^d^ CIE coordinates at the voltage of 8 V.

## Data Availability

Data are contained within the article. Further inquiries can be directed to the corresponding authors.

## Supplementary Materials

The following supporting information can be downloaded at https://www.mdpi.com/article/10.3390/mi17080940/s1, Table S1: Comparison of efficiency between device W5 and other non-doped white OLEDs [25,26,27,28,29,30].

## Abbreviations

The following abbreviations are used in this manuscript:

HAT-CN	Dipyrazino [2,3-f:2′,3′-h]quinoxaline-2,3,6,7,10,11-hexacarbonitrile
TAPC	1,1-Bis[(di-4-tolylamino) phenyl] cyclohexane, 4,4′-Cyclohexylidenebis [N, N-bis(4-methylphenyl) benzenamine]
B4PyPPM	4,6-Bis(3,5-di-4-pyridinylphenyl)-2-phenylpyrimidine
PO-01	1,3-Bis(carbazol-9-yl)benzen (e MCP) Iridium(III)bis(4-(4-tert-butylphenyl)thieno [3,2-c]-pyridinato-N,C2′)acetylacetonate
FIrpic	bis[(4,6-difluorophenyl)- pyridinato-N,C2′]c(picolinate)iridium(III)
