# Supplementary material for: Efficient Non-Doped White Organic Light-Emitting Diodes Based on an Interface Exciplex Structure
Source: Micromachines (Basel). 2026 Aug 7;17(8):940. doi: 10.3390/mi17080940 (PMC13515728; doi:10.3390/mi17080940)
Supplement: Supplementary file 1 [file micromachines-17-00940-s001.zip › micromachines-4468331-supplementary.pdf]

Table S1. Comparison of efficiency between device W5 and other non-doped white OLEDs.

**Device**	**V_{on}[a] (V)**	**CE_{max}[b] (cd/A)**	**PE_{max}[b] (lm/W)**	**EQE_{max}[b] (%)**
This work	2.7	30.59	32.03	11.27
[25]	3.0	20.5	13.6	10.9
[26]	-	31.0	24.3	12.6
[27]	2.65	30.3	28.8	18.7
[28]	-	15.5	12.8	6.72
[29]	2.6	44.9	50.4	23.4
[30]	2.4	63.3	83.2	19.6

[a]At the luminance of 1 cd/m^2; [b]The maximum efficiencies.
